# Antipyrin, &c.

**Published:** 1889-11-09

**Authors:** 


### ANTIPYRIN, &c.

This drug, which owes its conventional name to its power of reducing temperature in fevers, has of late fallen considerably in repute for this purpose, and at the same time has deservedly risen in estimation as a sedative of the nervous centres. Its effects in migraine are brilliant, but, like all new and good things, it has occasionally been overdone. It seems to us most useful in emergencies—we mean when two or three doses suffice to attain the end desired—and we should not advise its use in cases requiring continued administration for days or weeks, as, for example, in whooping-cough. Dr. Tuczek, however, not having the same scruples, gave his own son, a healthy boy of four years of age, suffering from a severe attack of whooping-cough, six-grain doses three times a day for three weeks. The child then vomited, became somnolent and comatose, and for three days exhibited epileptiform convulsions general and partial. The heart's action and respiration were irregular, the pulse slow and thready, the pupils dilated, and the temperature subnormal. There was acetonuria, and a macular exanthema. From this case, it would appear antipyrin is a cumulative poison. The child made a slow recovery.

